# Multi-species cryoEM calibration and workflow verification standard

**DOI:** 10.1107/S2053230X24010318

**Published:** 2024-10-31

**Authors:** Daija Bobe, Mykhailo Kopylov, Jessalyn Miller, Aaron P. Owji, Edward T. Eng

**Affiliations:** a Simons Electron Microscopy Center New York Structural Biology Center New York New York USA; Bristol-Myers Squibb, USA

**Keywords:** cryo-electron microscopy, single-particle analysis, benchmarking, calibration, resolution, workflow standard, training, education, 3D reconstruction and image processing, single-particle cryoEM, structure determination

## Abstract

Cryogenic electron microscopy (cryoEM) is a rapidly growing structural biology modality that has been successful in revealing molecular details of biological systems. However, unlike established biophysical and analytical techniques with calibration standards, cryoEM has lacked comprehensive biological test samples. Here, a cryoEM calibration sample consisting of a mixture of compatible macromolecules is introduced that can not only be used for resolution optimization, but also provides multiple reference points for evaluating instrument performance, data quality and image-processing workflows in a single experiment. This combined test specimen provides researchers with a reference point for validating their cryoEM pipeline, benchmarking their methodologies and testing new algorithms.

## Abstract 2

CryoEM technologies are emerging as a powerful structural science tool to reveal molecular details of biological systems, but have lacked comprehensive calibration standards for biomacromolecular workflow benchmarking. A cryoEM calibration sample mixture is introduced that may be used to evaluate more than the achievable information limit of the instrument by providing a workflow-validation and educational tool for biological samples.

## 1. Introduction

Cryogenic electron microscopy (cryoEM) has grown increasingly popular in structural biology as the quality and reliability of cryo-transmission electron microscopes have improved. However, due to the complex nature of the instrumentation, each microscope in operation is unique. Microscope builds can feature different gun sources, accelerating voltages, condenser systems, aberration correctors, energy filters and camera types. Each microscope is affected by its local environment, such as temperature, humidity, vibrations and electromagnetic fields, all of which influence data quality (Mills, 2021 ▸; Alink *et al.*, 2021 ▸). To ensure optimal performance, cryoEM practitioners rely on workflow-validation tests using an analytical benchmark standard (Kim *et al.*, 2018 ▸; Danev *et al.*, 2021 ▸; Gijsbers *et al.*, 2021 ▸).

Current requirements for cryoEM benchmarking standard are as follows.

*Accessibility*: can either be commercially purchased or has straightforward sample preparation with minimal maintenance requirements.

*Stability*: can be stored for prolonged periods of time without compromising structural integrity.

*Homogeneity*: minimal conformational and compositional heterogeneity.

*Reproducibility*: grid preparation can be standardized, reducing grid-to-grid variability.

Common benchmarking samples, including ribosomes, Tobacco mosaic virus, β-galactosidase, aldolase and apo­ferritin, meet the above criteria and have proven to be useful for resolution optimization (Bartesaghi *et al.*, 2018 ▸; Kim *et al.*, 2018 ▸; Yip *et al.*, 2020 ▸; Nakane *et al.*, 2020 ▸). Despite its importance, maximizing resolution is not the only goal of the cryoEM workflow. The data sets acquired for benchmarking resolution can also be used to support other aspects of the cryoEM pipeline such as pixel size calibration, neural network training, helical and single-particle processing, *etc*.

Here, we introduce a dedicated cryoEM calibration standard called the ‘EM ladder’, which extends beyond resolution benchmarking. A key aspect of the EM ladder is its ability to be used reproducibly under a wide variety of experimental conditions and pixel sizes for calibration, commissioning and certification of standard operating procedures. Similar to calibration standards for SDS–PAGE, size-exclusion chromatography and mass photometry, our calibration reagent is a mixture of components designed to be compatible with the full experimental workflow, rather than a single calibration reagent. We have combined a mixture of four samples: apoferritin (ApoF; *O* symmetry; ∼486 kDa, 20 kDa monomer), β-galactosidase (β-gal; *D*2 symmetry; ∼465 kDa, 116 kDa monomer), a virus-like particle (PP7; *I* symmetry; ∼3.4 MDa, 28 kDa monomer) and Tobacco mosaic virus (TMV; *H* symmetry; ∼40 MDa, 18 kDa monomer).

The focus of this calibration standard is to provide a stable and well characterized reference point that ensures that the full workflow of grid preparation, data collection, processing and analysis is reliable. As cryoEM technologies continue to evolve and be applied to new fields, this calibration standard broadens the scope where structural insights are not just high in resolution, but also high in accuracy, enabling the full potential of structural biological research.

## 2. Materials and methods

### 2.1. Samples

The stock apoferritin (human ferritin H chain; ApoF) was purified in 50 m*M* Tris pH 7.5, 100 m*M* NaCl, 0.5 m*M* TCEP by the Center on Membrane Protein Production and Analysis (COMPPÅ) at the New York Structural Biology Center. The original plasmid LF2422 contains human ferritin H chain cloned into pGEX2T with a TEV site instead of a thrombin site from the Protex facility at the University of Leicester and was a gift from Louise Fairall and Christos Savva. Appropriate commercial sources of cryoEM-ready apoferritin protein include horse spleen apoferritin (Sigma, catalog No. 178440), recombinant human apoferritin heavy and light chains (Scripps Laboratories, catalog No. F1021) or recombinant human apoferritin heavy chain (Thermo Scientific VitroEase Apoferritin Standard, catalog No. A51362).

Thyroglobulin (ThG) was purchased from Sigma (Product No. T1001). The lyophilized powder (40 mg) was reconstituted in a storage buffer consisting of 20 m*M* HEPES pH 7.4, 150 m*M* NaCl.

β-Galactosidase (β-gal) was purchased from Sigma (Product No. G5635). The lyophilized β-gal (50 mg) was reconstituted in a storage buffer consisting of 50 m*M* Tris–HCl, 10 m*M* MgCl_2_, 10 m*M* β-mercaptoethanol pH 7.3.

PP7 virus-like particles (VLPs) were a gift from M. G. Finn’s group at Georgia Institute of Technology. PP7 wild-type sample was provided as a 1 mg ml^−1^ solution in 100 m*M* phosphate-buffered saline (PBS) pH 7.0 as described previously (Zhao *et al.*, 2019 ▸). The current authors have the permission of M. G. Finn to distribute PP7 VLP samples upon request.

Tobacco mosaic virus (TMV) at a stock concentration of 34.85 mg ml^−1^ in Tris-buffered saline (TBS) was a gift from Ruben Diaz-Avalos at La Jolla Institute for Immunology. TMV is commercially available from ATCC (PV-599).

For the final mixture that was used for cryoEM imaging, each protein was diluted with 50 m*M* HEPES pH 7.5, 100 m*M* NaCl. This buffer was chosen for its compatibility with each protein. The final concentration of each protein prior to mixing was as follows: 0.16 mg ml^−1^ apoferritin, 0.10 mg ml^−1^ PP7, 1 mg ml^−1^ β-gal, 0.17 mg ml^−1^ TMV. To create the final mixture, 2 µl of each protein were added together. The concentrations of each protein in the final mixture were as follows: 0.04 mg ml^−1^ ApoF, 0.025 mg ml^−1^ PP7, 0.25 mg ml^−1^ β-gal, 0.0425 mg ml^−1^ TMV. The mixture was aliquoted, snap-frozen and stored at −80°C for later use.

### 2.2. Negative-stain grid preparation

Continuous carbon grids made in-house were plasma-cleaned with a hydrogen/oxygen mixture for 30 s on a Gatan Solarus. Two 20 µl droplets of distilled water were added to Parafilm followed by three 20 µl droplets of 2% uranyl formate. 3 µl of sample was applied to the continuous carbon grid for 45 s to 1 min. The grid was side-blotted, followed by a sequence of dipping into a droplet carbon-side down and side-blotting for both water droplets and two uranyl formate droplets. The grid was held in the last uranyl formate droplet for 1 min before side-blotting and back-blotting to remove excess stain.

### 2.3. CryoEM grid preparation

UltrAuFoil R1.2/1.3 300 mesh holey gold grids (Quantifoil Micro Tools, Grosslöbichau, Germany) were plasma-cleaned with an argon/oxygen mixture for 7 s on a Gatan Solarus. 3 µl of freshly thawed protein from the final mixture was applied to the UltrAuFoil grid, blotted for 2.5 s after a 30 s wait time and then plunge-frozen in liquid ethane, cooled by liquid nitrogen, using a Vitrobot Mark III (FEI, Hillsboro, Oregon, USA) at 75% relative humidity.

### 2.4. Imaging

#### 2.4.1. Screening

For screening, a Thermo Fisher Scientific Tecnai 12 with a TVIPS F416 CMOS camera was operated at 120 kV with a 100 µm objective aperture. Images were collected at a pixel size of 2.46 Å using *Leginon* (Suloway *et al.*, 2005 ▸) at 800 ms per exposure with a dose of ∼50 e^−^ Å^−2^ and a nominal defocus range of 2–4 µm.

#### 2.4.2. Data acquisition

For final data acquisition, a Thermo Fisher Scientific Titan Krios G2 with a spherical aberration corrector and a post-column Gatan Image Filter (GIF) and Gatan K2 Summit was operated at 300 kV with a 70 µm C2 aperture and a 100 µm objective aperture. Images were collected in counting mode with a 20 eV slit width and a calibrated pixel size of 1.096 Å using *Leginon* (Suloway *et al.*, 2005 ▸) at a dose rate of 6.95 e^−^Å^−2^ s^−1^ with a total exposure of 10 s, for an accumulated dose of 69.46 e^−^ Å^−2^. A total of 3996 images were collected at a nominal defocus range of 1.5–2.5 µm. Given data-retention policies, the original movies were not archived and the *MotionCor*2 with dose weighting (Zheng *et al.*, 2017 ▸) aligned summed images from the *Appion* (Lander *et al.*, 2009 ▸) pre-processing pipeline were stored as JPEG files for *Appion* image-viewer functionality (Eng *et al.*, 2019 ▸). For this study, these images were converted back to 32-bit MRC files using *EMAN*2 (Tang *et al.*, 2007 ▸) for further processing in *cryo­SPARC* (Punjani *et al.*, 2017 ▸).

### 2.5. Image processing

**Figure 1:**
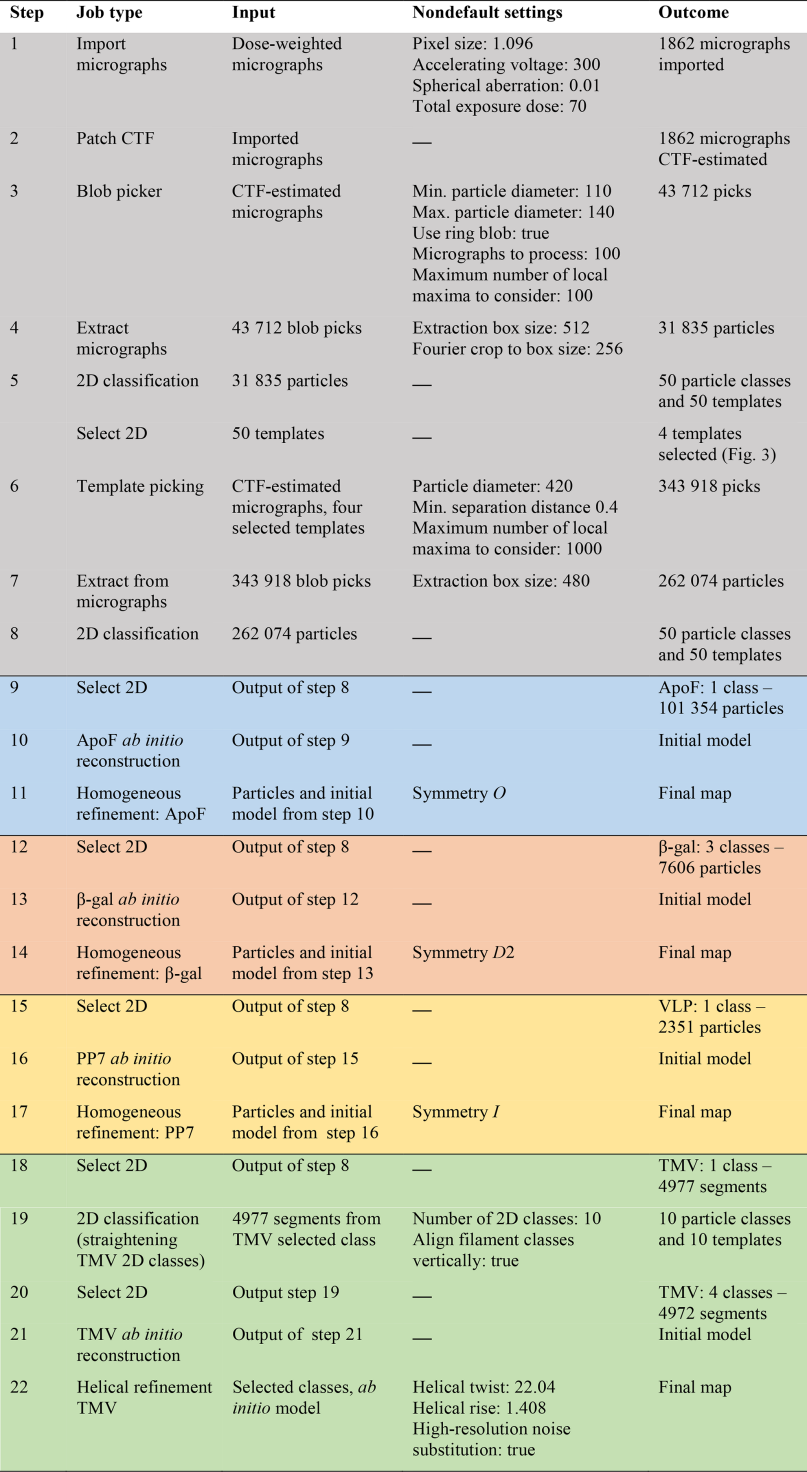
*CryoSPARC* processing parameters for one-shot parallel processing. Gray rows, processing common to all four species; blue rows, apoferritin-specific steps; red rows, β-gal-specific steps; yellow rows, PP7-specific steps; green rows, TMV-specific steps. See also Supplementary Fig. S7.

The data set was manually curated and 1862 images were selected from the 3996 images. The only selection criterion was the presence of three or more protein types in the micrograph. After conversion to 32-bit MRC files, selected images were imported into *cryoSPARC* as micrographs for CTF estimation, particle picking and extraction, and subjected to 2D and 3D classification, initial model generation and refinement. Processing settings for the one-shot processing are reported in Fig. 1 ▸ with all nondefault settings highlighted. Additional details and intermediate results of the processing of individual components are provided in the supporting information.

### 2.6. Pixel size calibration

Pixel size calibration was performed against a recently deposited ApoF model (PDB entry 8f4l; H. Shi, C. Wu & X. Zhang, unpublished work). The ApoF map generated in this study (EMDB entry EMD-41923) was opened in *UCSF Chimera* version 1.13.1 and displayed at a 1.635 threshold. The ApoF model (PDB entry 8f4l) was rigid-body fitted into the map density using the ‘Fit in Map’ tool from the Volume tools. The map’s pixel size varied from 1.104 to 1.081 Å, the model fit was updated for each pixel size and the number of atoms outside the map was recorded and plotted in *Excel* (Supplementary Fig. S6).

## 3. Results and discussion

**Figure 2:**
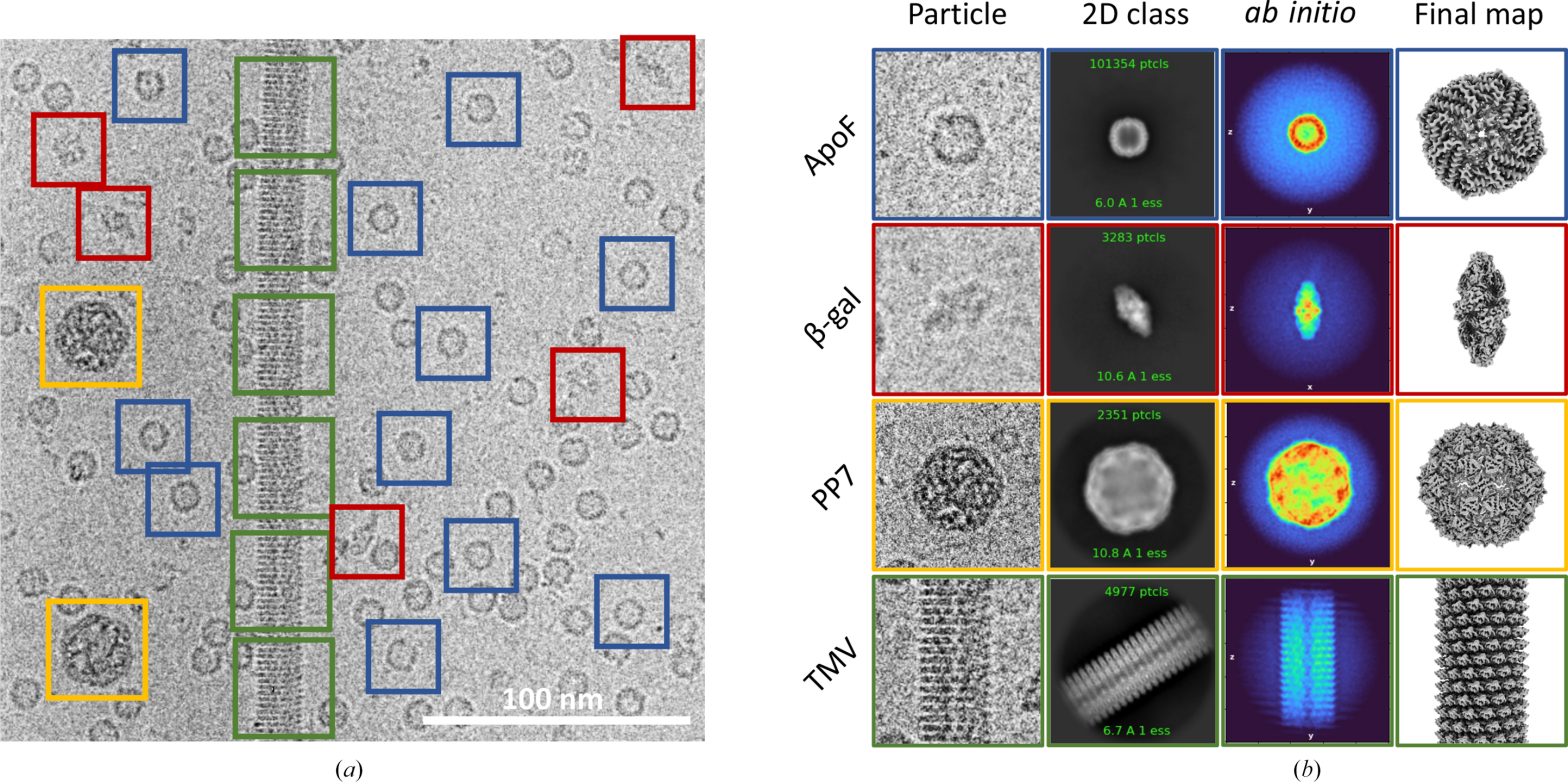
Multi-species data-set overview. (*a*) An exemplar micrograph with four types of particles boxed out (ApoF, blue; β-gal, red; PP7, yellow; TMV, green). (*b*) Typical processing results: individual extracted particles, 2D class averages, *xy* slice through the center of *ab initio* reconstruction and 3D map visualized in *ChimeraX*.

Four components were chosen for the EM ladder: ApoF, β-gal, PP7 and TMV (Fig. 2 ▸; Table 1 ▸). ApoF is an octahedral protein cage and is a widely used specimen for characterization of the resolution limit of cryoEM microscopes. Mouse ApoF has yielded the highest available resolution from cryoEM single-particle analysis. Commercially available horse spleen ApoF (Sigma, Product No. 178440) can be used, resulting in ∼2 Å reconstructions (Nakane *et al.*, 2020 ▸; PDB entry 6pxm; M. Kopylov, K. Kelley, L. Y. Yen, W. J. Rice, E. T. Eng, B. Carragher & C. S. Potter, unpublished work). However, ApoF is notoriously difficult to reconstruct from poor-quality data acquired from thick ice, charge-coupled detectors or low-voltage microscopes (Neselu *et al.*, 2023 ▸; Henderson & McMullan, 2013 ▸; Russo & Passmore, 2014 ▸), so β-gal serves as a low-symmetry (*D*2) standard in this EM ladder mixture. It is commercially available and has been used extensively as a high-resolution test specimen. Prior to vitrification, β-gal can be incubated with various ligands, such as phenylethyl β-d-thiogalactopyranoside (PETG), for additional stability (Bartesaghi *et al.*, 2018 ▸). β-gal can be replaced in this mixture by aldolase (Sigma, Product No. A2714), conalbumin (Sigma, Product No. C0755) or any other low-symmetry small protein, although the particle concentration and ratio of the mixture may differ. PP7 VLPs are ∼22 nm icosahedral particles that can be reconstructed in a broad resolution range from 30 to 2.5 Å. PP7 particles are twice the diameter of ApoF and can serve as an alternative to ApoF as a pixel size calibration standard. PP7 VLPs can be replaced with other large particles such as adeno-associated virus, bacteriophage Qβ, proteasome or GroEL (Sigma, Product No. C7688). Finally, TMV has been extensively used as a resolution test specimen. It can be effectively used as another alternative for pixel size calibration and for processing as it provides a good starting point in learning helical processing. TMV can be replaced with similar helical viruses, microtubules (Cytoskeleton MT001 or MT002) or other helically symmetric elongated constructs. These samples have been routinely used to benchmark various steps of the cryoEM workflow individually, but have not previously been used in combination.

After negative-stain screening of the initial mixture, the concentration of ApoF was halved due to it being the dominant protein in the micrographs (Supplementary Fig. S1). Each mixture was subsequently changed based on the amount of each protein seen during screening. The decision to switch to cryo screening was made after the observation that a mixture that had a good distribution of each protein in negative stain did not have the same distribution in cryo screening. During cryo screening, broken ends of thyro­globulin (ThG) were visible in the background of the micrographs. ThG requires a detergent, such as CHAPS, to remain fully intact in cryo screening. In further mixtures, ThG was switched to β-gal so that all proteins in the mixture could be fully reconstructed without adding detergent to the buffer.

Although ApoF has become a widely used benchmarking standard for high-resolution testing of all transmission electron microscopes (Chan *et al.*, 2024 ▸; Sun *et al.*, 2021 ▸; Peng *et al.*, 2023 ▸), it still presents a challenge when processing. Due to the comparable size of β-gal and ApoF, having both present within a micrograph is useful to test several particle pickers for their ability to differentiate between the two species, while also being able to benchmark the microscope. In contrast to ApoF, VLPs are larger, simple to pick and can be used for low-resolution testing as well as calibrating pixel sizes. In addition to the single-particle processing practice, including TMV in the mixture presents the opportunity to learn or improve the ability to perform helical processing. TMV has been widely studied, so knowing the pitch, rise and twist is helpful whether one is processing from scratch and needing to confirm parameters or is just trying to learn the different steps involved in helical processing versus single-particle processing.

**Figure 4:**
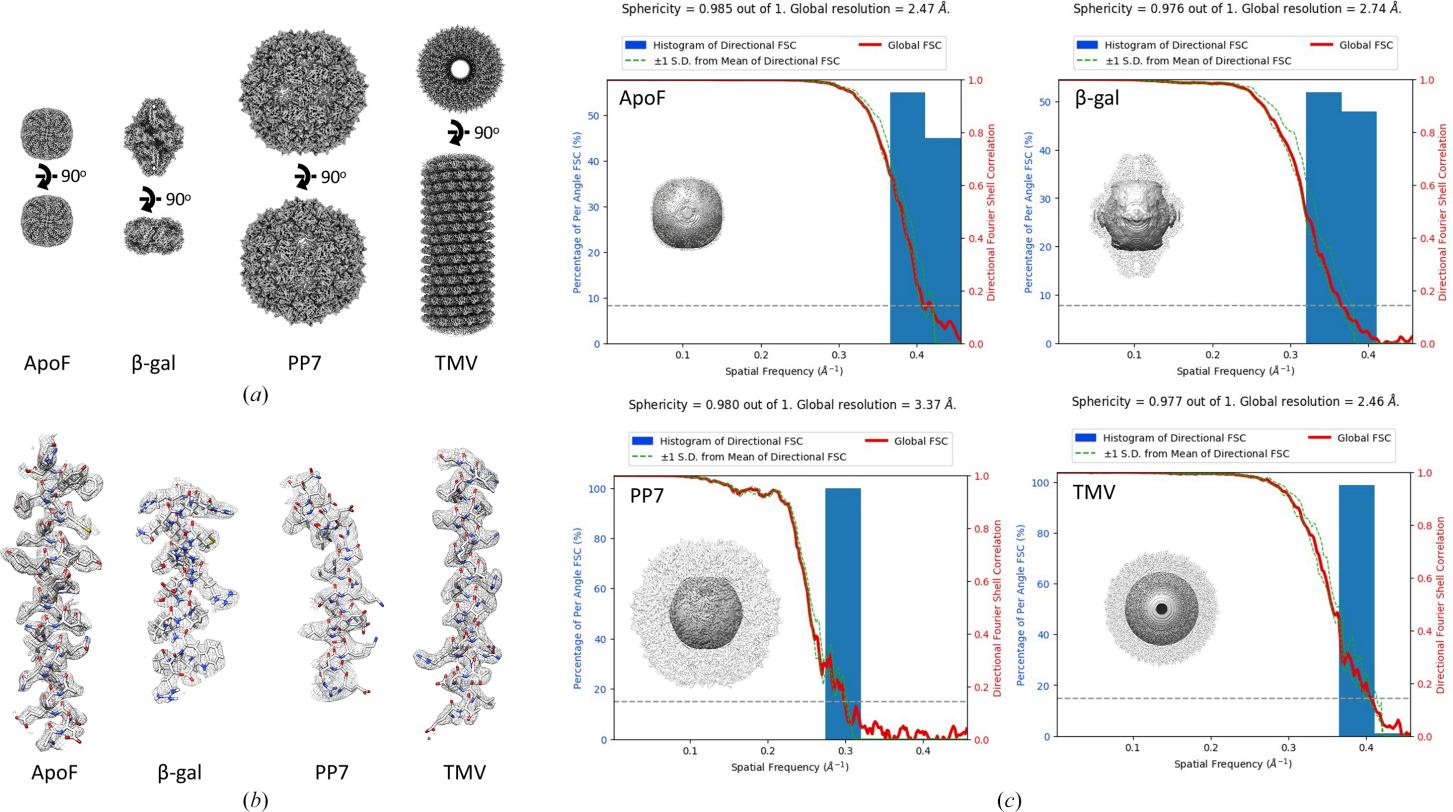
CryoEM reconstructions from a multi-species data set. (*a*) Isosurface representations of (left to right) ApoF, β-gal, PP7 and TMV. (*b*) PDB model fitted into a mesh map of (left to right) ApoF (PDB entry 1fha chain *A* residues 14–42), β-gal (PDB entry 6x1q chain *A* residues 429–448), PP7 (PDB entry 1dwn chain *A* residues 96–121) and TMV (PDB entry 6r7m chain *A* residues 107–136. (*c*) Histogram and directional FSC plot with sphericity representation and transparent isosurface view of (left to right) ApoF at a global resolution of 2.47 Å with 164 200 particles and a sphericity of 0.985, β-gal at a global resolution of 2.74 Å with 37 617 particles and a sphericity of 0.976, PP7 at a global resolution of 3.37 Å with 1803 particles and a sphericity of 0.98 and TMV at a global resolution of 2.46 Å with 12 038 particles and a sphericity of 0.977.

A total of 3996 micrographs of the EM ladder were acquired. Despite multiple trials to obtain an even distribution of the different particle types (Supplementary Fig. S1), not all micrographs contained all four different proteins. This variation may in part be due to the differential preferences of this diverse set of particles for a specific ice thickness and interaction with the air–water interface. To enrich in multi-species micrographs, each micrograph was manually selected as containing at least three species in the field of view. All four species can be easily identified in a micrograph: ApoF ‘donuts’, small spindle-shaped β-gal, large hexagons of PP7 VLPs and ‘train tracks’ of TMV (Fig. 2 ▸*a*). Processing these data also provides clear and easily interpretable results from particle picking, through 2D classification and initial model generation, to 3D refinement (Fig. 2 ▸*b*). Despite the varied sizes and symmetry types present in this data set, initial processing can be performed in only 22 *cryoSPARC* jobs following a one-shot processing strategy, where a single box size is used for all particles (Figs. 1 ▸ and 3 ▸). This results in sub-3 Å reconstructions for two species: the most abundant ApoF and helical TMV. Particle picking, box size, and classification and refinement strategies can be optimized from this point (Supplementary Fig. S2–S5). Final reconstructions for this multi-species data set resulted in GSFSC resolutions of 2.47 Å for ApoF, 2.74 Å for β-gal, 3.37 Å for PP7 and 2.46 Å for TMV (Fig. 4 ▸).

The ApoF map was used to re-estimate the microscope pixel size, which was found to be 1.094 Å, a ∼0.2% difference from the previously calibrated pixel size for this microscope (Supplementary Fig. S6). This calibration is only accurate if the originally deposited model was accurate. Therefore, it can be a good starting point for pixel size validation; however, other methods should be used to verify it, such as the use of calibration grids or gold diffraction (Dickerson *et al.*, 2024 ▸).

Sample preparation and grid vitrification are crucial steps in the production of data sets yielding high-resolution structures. The behavior of particles in the thin aqueous film produced during grid blotting is a driving factor in contemporary cryoEM grid production, often dictating the ice thickness in which particles reside and limiting the attainable resolution of their respective reconstructions. While some macromolecular targets behave well with little optimization, others require thorough optimization of biochemical purification and grid-production parameters to obtain grids that are amenable to high-resolution structure determination. In this study, four relatively well behaved samples were combined on a single grid, which required moderate optimization efforts to obtain micrographs containing all four proteins with a thin enough sample thickness to enable structure determination. While minimizing ice thickness to maximize resolution was not our goal, this remains a possible future direction, as high-resolution reconstruction is a major goal for hardware testing and workflow optimization. Although outside the purview of this study, it may be of interest to assess the behavior of particle mixtures on the grid with respect to the ice thickness in which they reside and to determine whether the inclusion of different protein types can alter this ice-thickness window. Since single-protein data sets exist for these four well characterized cryoEM test specimens, comparison of this data set with corresponding Electron Microscopy Image Archive (EMPIAR) data sets for ice thickness and attainable resolution may be of interest. As apoferritin is a common specimen for hardware testing and pixel size calibration, its inclusion in grids containing target specimens could have utility in enabling hardware validation and pixel size calibration for every session. Our experience in optimizing particle concentrations for multi-protein micrographs suggests that apo­ferritin tends to dominate on grids with protein mixtures, so this strategy would likely add another layer of optimization to samples for a relatively modest benefit.

As new practitioners adopt cryoEM workflows in their research programs, they need guidelines not only to ensure that their pipelines are compatible with their experimental requirements, but also to be confident that their instrumentation can yield reliable and accurate results. A driving factor for the choice of samples for this study is the selection of a molecular-dimension range that would cover a size range larger than the experimental samples used in a typical cryoEM study. Mixtures that are optimized for different properties may be useful; for example, a low- or high-molecular-weight standard to ensure that the specimens are not only biocompatible, but are also contained within a reproducible sample thickness during production. The core goal for whichever mixture is chosen is a cryoEM calibration standard that can validate a particular imaging condition within a single experiment and generate training data sets that can be used for benchmarking and cryoEM education.

## 4. Conclusions

The introduction of dedicated cryoEM calibration standards extends far beyond benchmarking instrumentation by providing baseline control experiments for researchers to standardize their entire workflow for use in biophysical and structural biology research. The EM ladder can be used for high-resolution reconstructions, but the main goal is to ensure the quality and reproducibility of data. By combining multiple specimens in a single experimental sample, an array of desired properties, features and/or criteria of interest may be tested. Furthermore, this standardization endeavor extends to imaging settings, offering a reliable foundation for electron microscopes to consistently cater to a diverse array of biological samples. As cryoEM methodologies continue to evolve, control experiments form a necessary foundation of consistency and precision for integrative analysis. Taken together, the cryoEM calibration standard concept facilitates cross-disciplinary structural biology methodologies that rely on the reproducibility of cryoEM data.

## Supplementary Material

EMDB reference: PP7 virus-like particles, EMD-41917

EMDB reference: β-galactosidase, EMD-41919

EMDB reference: human apoferritin, EMD-41923

EMDB reference: Tobacco mosaic virus, EMD-41924

Supplementary Figures. DOI: 10.1107/S2053230X24010318/rf5044sup1.pdf

## Figures and Tables

**Figure 3:**
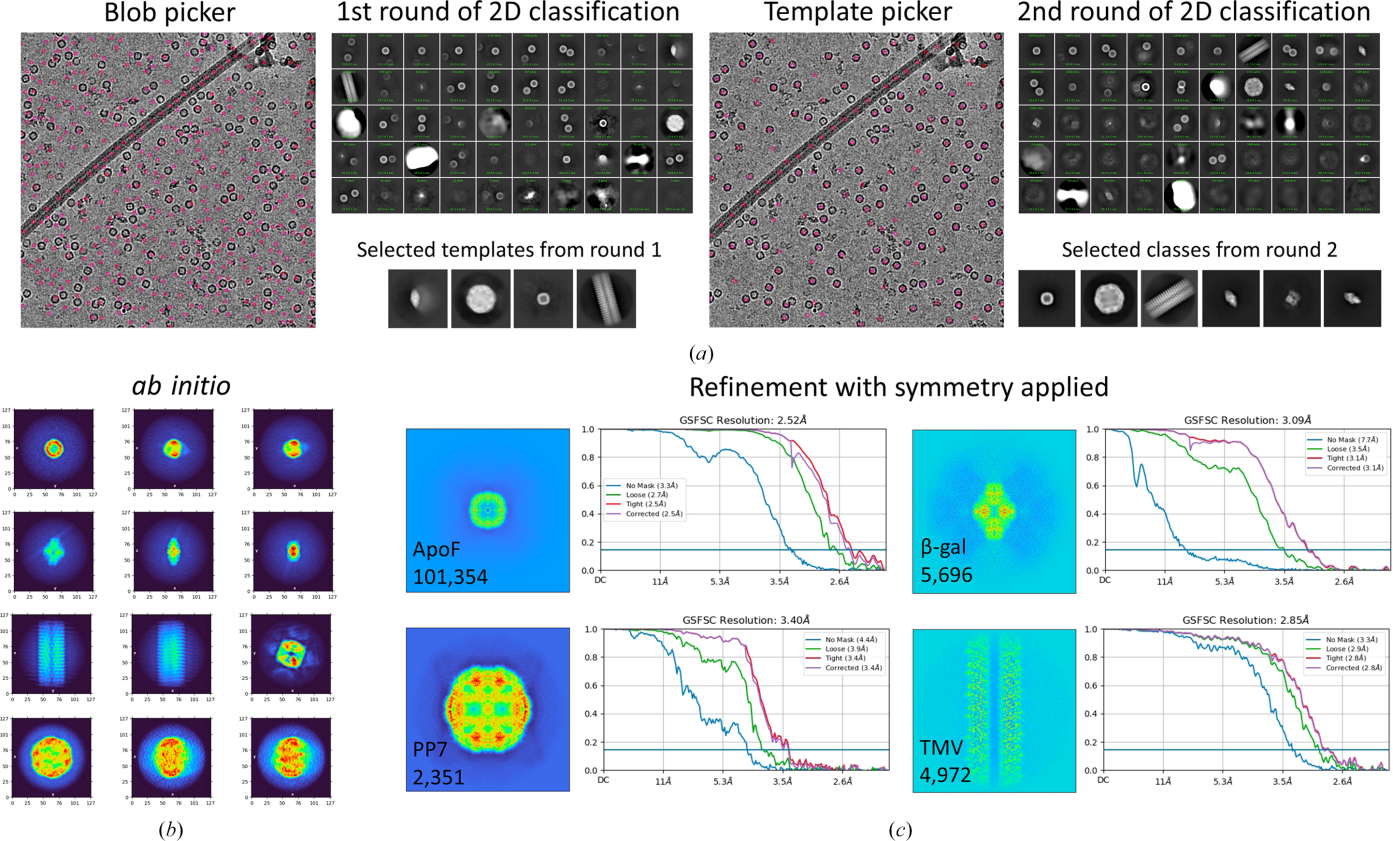
One-shot processing strategy in* cryoSPARC*. (*a*) Diagnostic images from two rounds of particle picking and 2D classification. Blob picking was first used, and templates were selected for the second round of picking using a template picker. (*b*) Particles belonging to classes corresponding to ApoF, β-gal, PP7 or TMV were selected and used as input for individual *ab initio* jobs with *K* = 1. (*c*) Output of 3D refinement jobs for all four species. Step-by-step information on jobs and settings for one-shot processing in *cryoSPARC* is described in Fig. 1 ▸.

**Table 1:** Protein composition mixture trials with representative images in Fig. 3 ▸ Stock concentrations and final concentrations after mixing (in parentheses) are in mg ml^−1^.

Mixture No.	TMV	PP7	ApoF	ThG	β-gal
1	0.17 (0.0425)	0.05 (0.0125)	0.08 (0.02)	0.1 (0.025)	—
2	0.17 (0.0425)	0.05 (0.0125)	0.004 (0.001)	0.1 (0.025)	—
3	0.17 (0.0425)	0.05 (0.0125)	0.00008 (0.00002)	1 (0.25)	—
4	0.17 (0.0425)	0.05 (0.0125)	0.00008 (0.00002)	0.1 (0.025)	—
5	0.17 (0.0425)	0.05 (0.0125)	0.0004 (0.0001)	0.1 (0.025)	—
6	0.17 (0.0425)	0.05 (0.0125)	0.04 (0.01)	0.1 (0.025)	—
7	0.17 (0.0425)	0.05 (0.0125)	0.08 (0.02)	0.1 (0.025)	—
8	0.17 (0.0425)	0.05 (0.0125)	0.08 (0.02)	—	1 (0.25)
9	0.17 (0.0425)	0.1 (0.025)	0.08 (0.02)	—	1 (0.25)
10†	0.17 (0.0425)	0.1 (0.025)	0.16 (0.04)		1 (0.25)

† Mixture 10 was used for the studies shown in Figs. 2 ▸, 3 ▸ and 4 ▸. The final mixture composition was 1.1 n*M* TMV, 5 n*M* PP7, 87 n*M* ApoF and 526 n*M* β-gal.

## Data Availability

The cryoEM maps of all four components processed following the workflows described here have been deposited in the Electron Microscopy Data Bank (EMDB) with the following accession codes: human apoferritin, EMD-41923; β-galactosidase, EMD-41919; PP7 virus-like particles, EMD-41917; Tobacco mosaic virus, EMD-41924. Electron micrographs have been made available in the Electron Microscopy Image Archive (EMPIAR) with accession code EMPIAR-11693.
