# Supplementary material for: Multi-species cryoEM calibration and workflow verification standard
Source: Acta Crystallogr F Struct Biol Commun. 2024 Oct 31;80(Pt 11):320–7. doi: 10.1107/S2053230X24010318 (PMC11533365; doi:10.1107/S2053230X24010318)
Supplement: Supplementary file 1 [file f-80-00320-sup1.pdf]

STRUCTURAL BIOLOGY
COMMUNICATIONS

Volume 80 (2024)

Supporting information for article:

Multi-species cryoEM calibration and workflow verification standard

Daija Bobe, Mykhailo Kopylov, Jessalyn Miller, Aaron P. Owji and Edward T. Eng

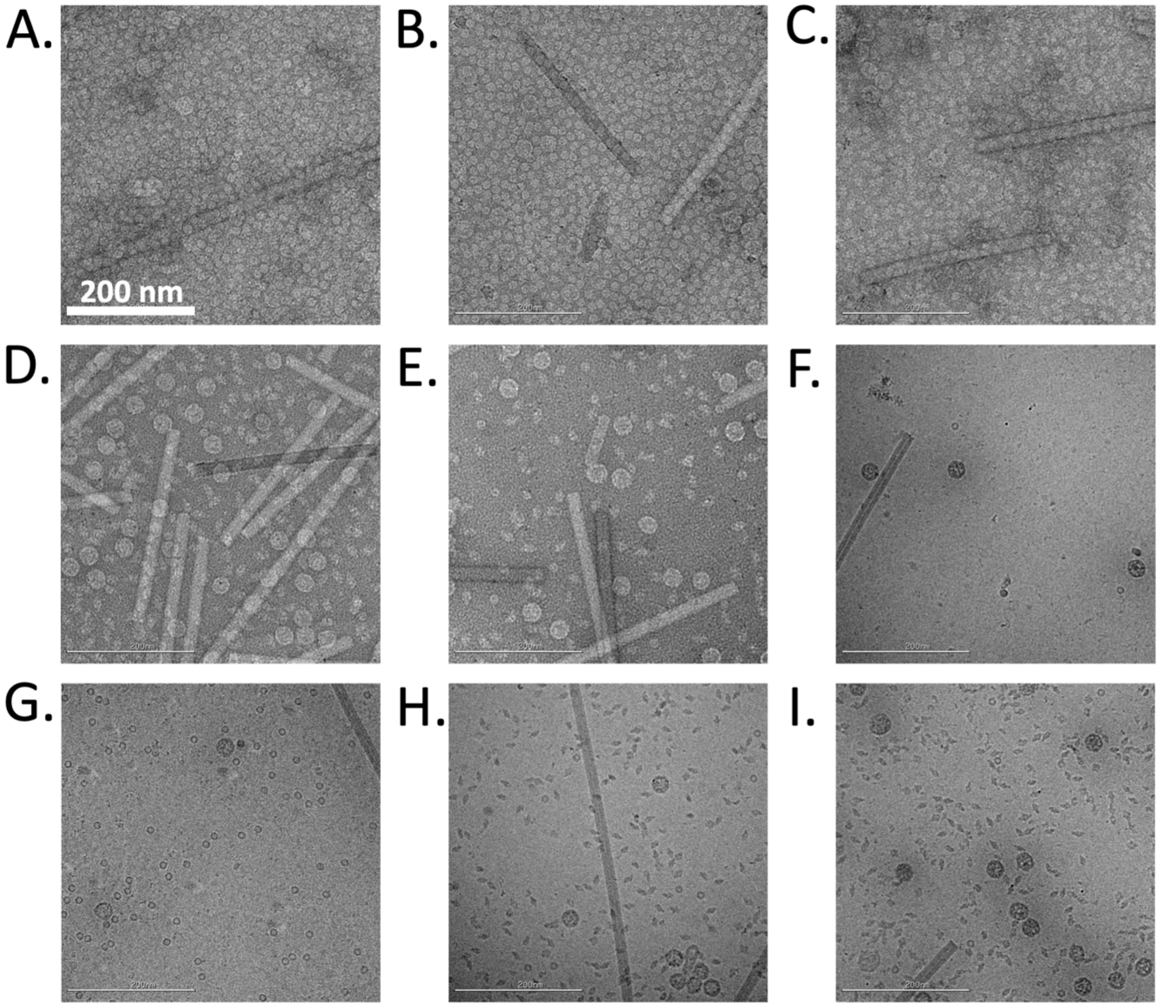

Figure S1 Representative images from trials of multi-protein mixtures. A through E: negative stain micrographs of mixes 1 through 5. F through I: cryo micrographs of mixes 6 through 9. A=mix 1, B=mix 2, C=mix 3, D=mix 4, E=mix 5, F=mix 6, G=mix 7, H=mix 8, I=mix 9. Exact mix compositions are shown in Table 2.

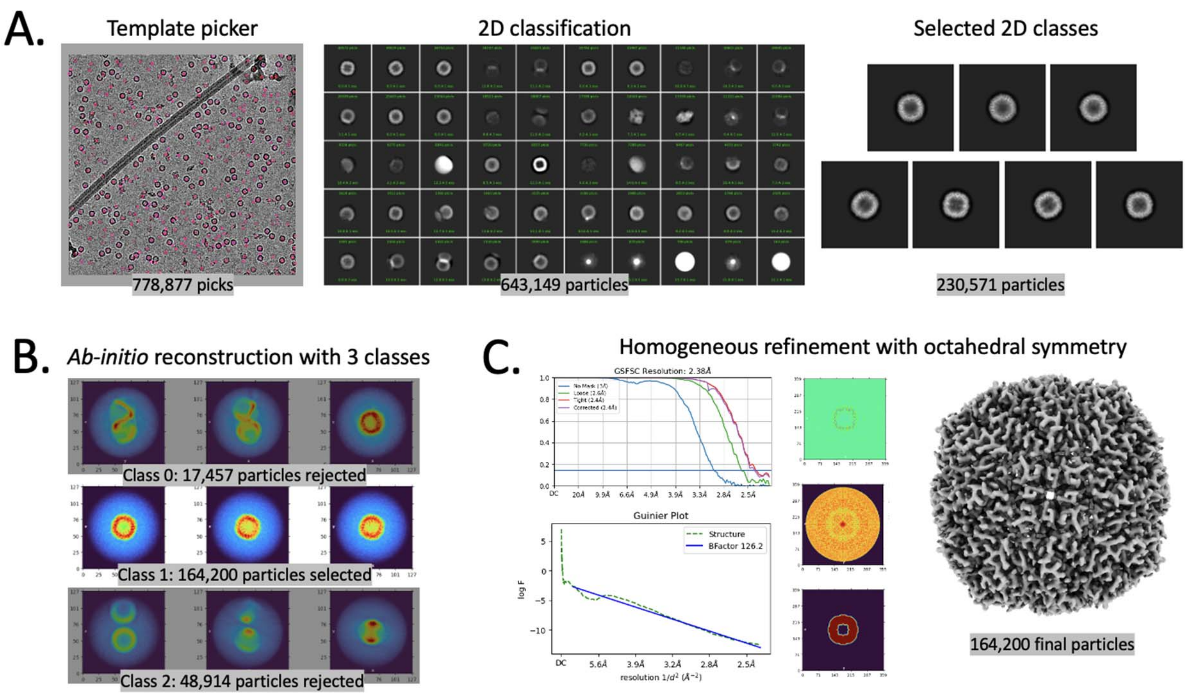

Figure S2 ApoF processing. ApoF template from one-shot processing was used for template picking. A. Workflow steps include Template picker, 2D classification and Select 2D. B. *Ab-initio* reconstructions using 3 classes. C. Homogeneous refinement with symmetry applied.

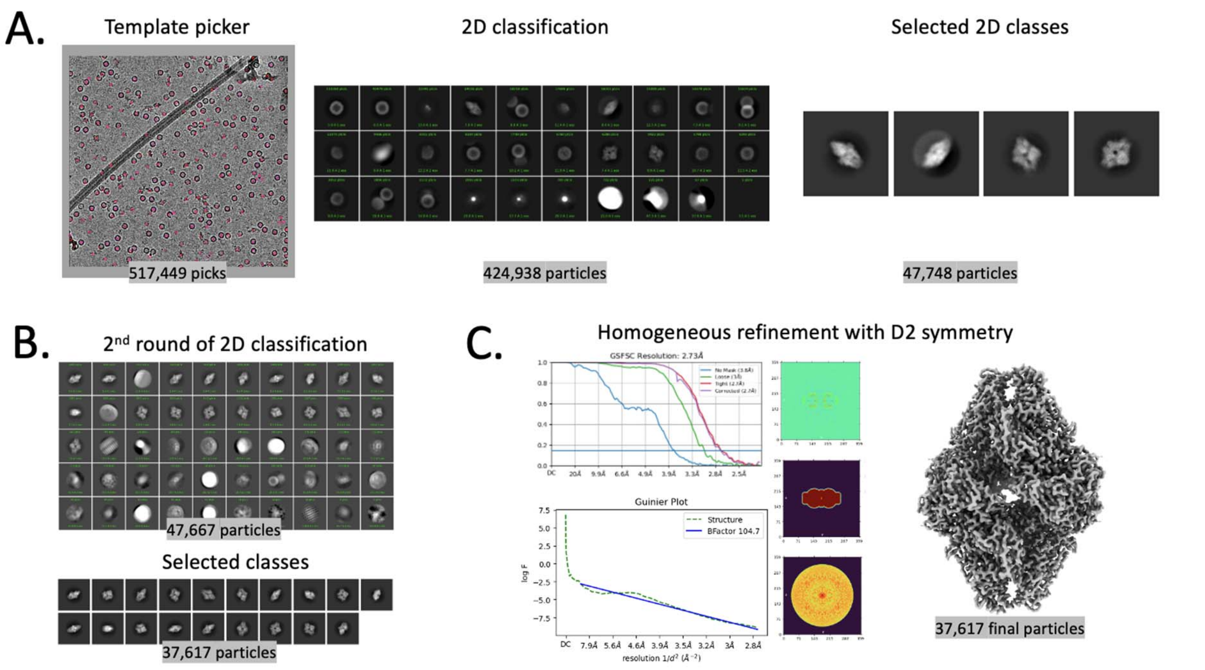

Figure S3 β-gal processing. A. Workflow steps include Template picker, 2D classification and Select 2D. B. *Ab-initio* reconstructions using 3 classes. C. Homogeneous refinement with symmetry applied.

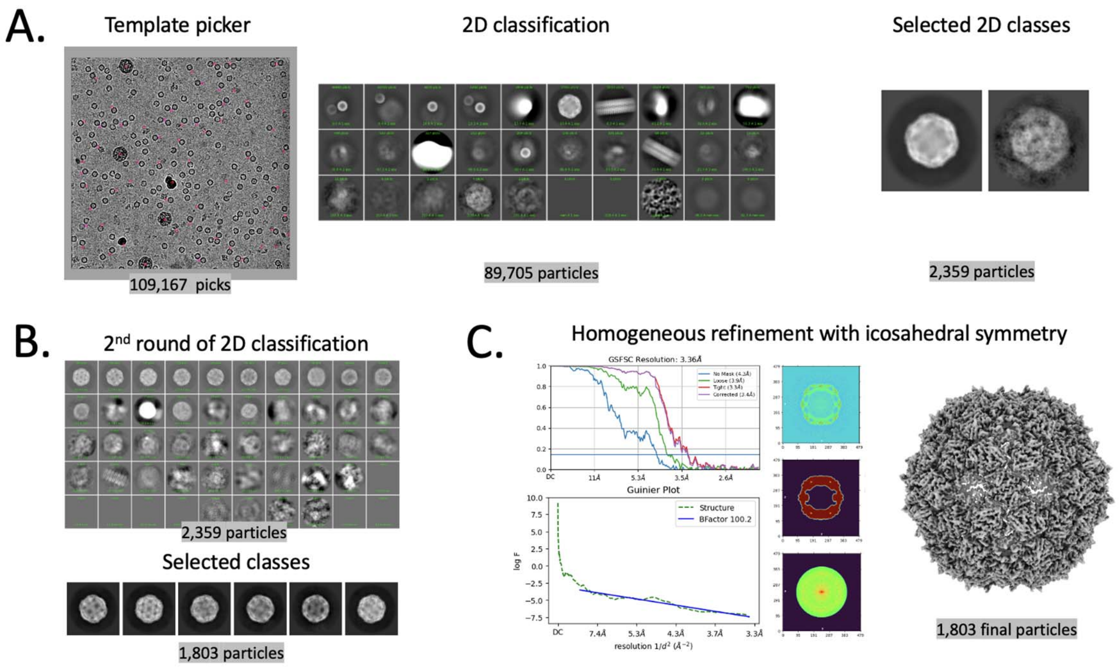

Figure S4 VLP processing. A. Workflow steps include Template picker, 2D classification and Select 2D. B. *Ab-initio* reconstructions using 3 classes. C. Homogeneous refinement with symmetry applied.

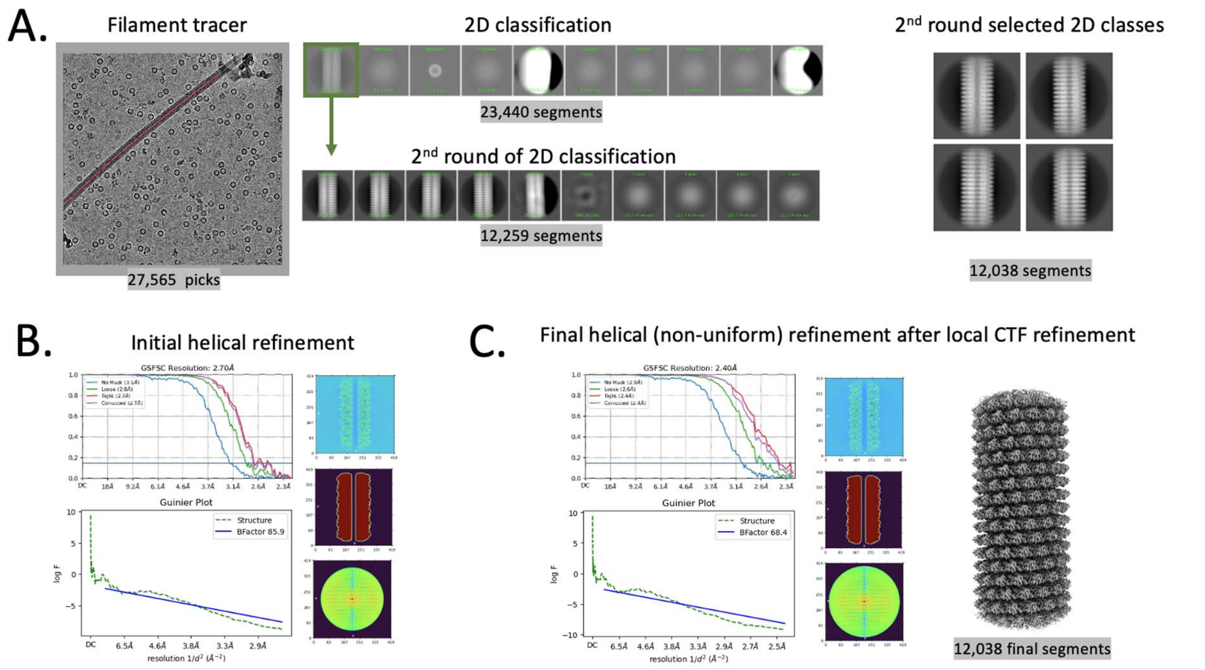

Figure S5 TMV processing. A. Workflow steps include Template picker, 2D classification and Select 2D. B. *Ab-initio* reconstructions using 3 classes. C. Homogeneous refinement with symmetry applied.

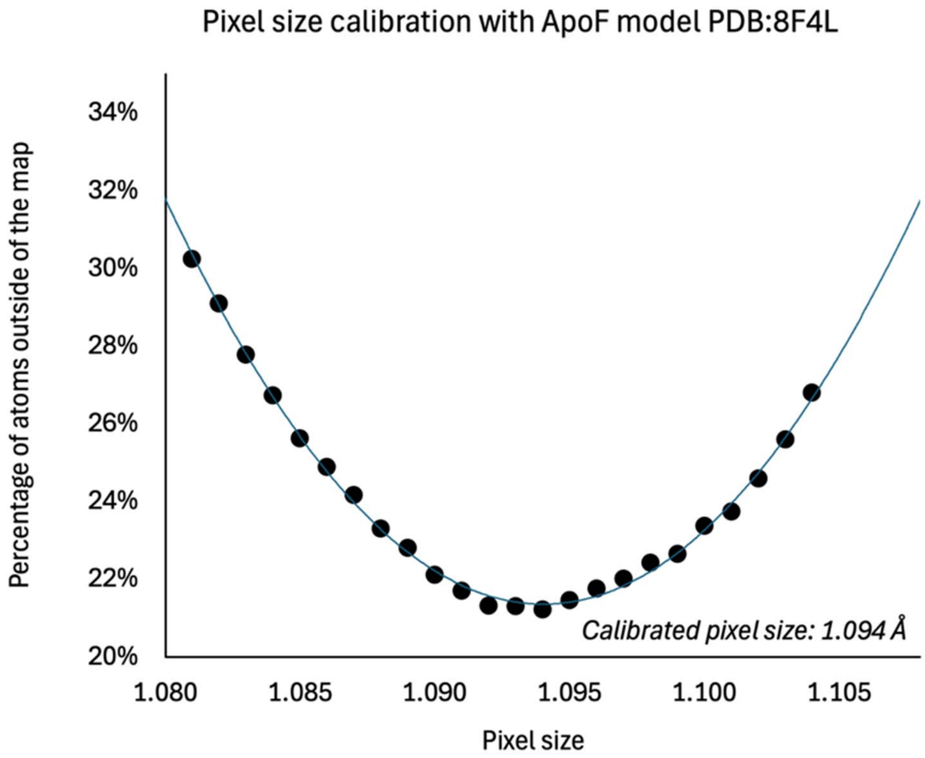

Figure S6 Pixel size calibration with ApoF model PDB:8F4L. Pixel size calibration was performed using the "Model fit" tool in Chimera with the deposited ApoF map using a threshold of 1.635. The pixel size was varied in increments of 0.001 Å. The resulting calibrated pixel size from this dataset was 1.094 Å vs. expected 1.096 Å.

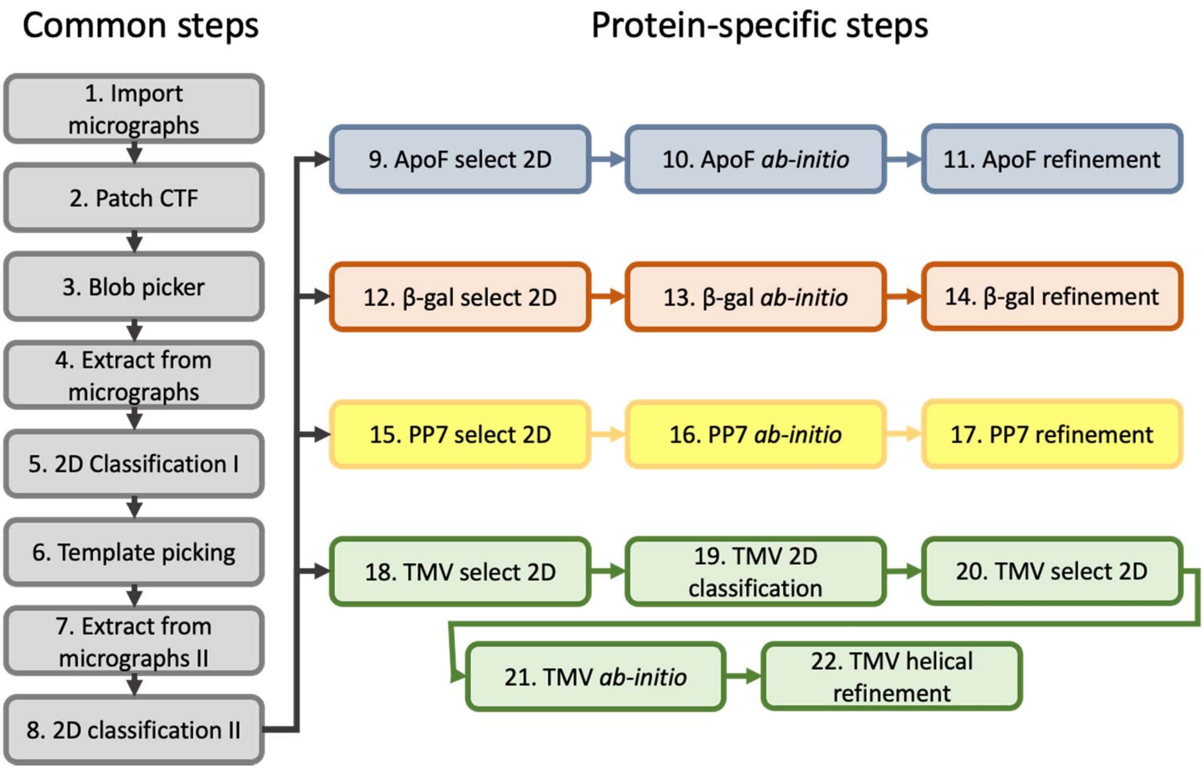

Figure S7 One-shot parallel processing at-a-glance (step details are in Table 1). Processing strategy overview for the multi-species dataset. The goal is to obtain an initial sample assessment and refinement maps in minimal steps. Exact job parameters are described in Table 1.
